# The Rubber Contest

**Published:** 1868-02

**Authors:** 


### THE RUBBER CONTEST.

The cases pending in the U. S. District Courts in Cincinnati and Chicago, were argued the last of February, and submitted to the Courts. No decision has yet been rendered, and may not be for some time to come.

We would suggest to our professional brethren to be patient till these decisions are rendered. We will try and stand it if they do not come for six months. We should be pleased to publish a brief of our counsel's argument in this number, yet space forbids it; perhaps we may in our next issue. T.

